# Identification and validation of autophagy-related genes during osteogenic differentiation of bone marrow mesenchymal stem cells

**DOI:** 10.22038/IJBMS.2022.65528.14420

**Published:** 2022-11

**Authors:** Yan Li, Xiu Yao, Yanjun Lin, Yifeng Xing, Chaowei Liu, Jianghan Xu, Dong Wu

**Affiliations:** 1 Provincial Engineering Research Center of Oral Biomaterial, Fujian Medical University, Fuzhou, Fujian, 350001, China; 2 Department of Oral Implantology, School and Hospital ​of Stomatology, Fujian Medical University, Fuzhou, Fujian, 350001, China; 3 Research Center of Dental and Craniofacial Implants, Fujian Medical University, Fuzhou, Fujian, 350001, China; 4 Department of Implantology, Shanghai Stomatological Hospital and School of Stomatology, Fudan University, Shanghai, 200433, China; # These authors contributed eqully to this work

**Keywords:** Autophagy, Bioinformatics, Bone marrow mesenchymal - stem cells, Bone regeneration, Osteogenesis

## Abstract

**Objective(s):**

Osteogenic differentiation of bone marrow mesenchymal stem cells (BMSCs) is an essential stage in bone formation. Autophagy plays a pivotal role in the self-renewal potential and pluripotency of stem cells. This study aimed to explore the function of autophagy-related genes during osteogenic differentiation of BMSCs.

**Materials and Methods:**

The differentially expressed autophagy-related genes (ARGs) were obtained from the GEO and HADb databases. The Gene Ontology (GO) and Kyoto Encyclopedia of Genes and Genomes (KEGG) enrichment analyses were performed using R software. The PPI and hub gene mining networks were constructed using the STRING database and Cytoscape. Finally, the RT-qPCR was conducted to validate the expression level of ARGs in BMSCs.

**Results:**

Thirty-seven differentially expressed ARGs were finally obtained, including 12 upregulated and 25 downregulated genes. GO and KEGG enrichment analysis showed that most of these genes were enriched in apoptosis and autophagy. The PPI network revealed strong interactions between differentially expressed ARGs. The expression level of differentially expressed ARGs tested by RT-qPCR showed 6 upregulated ARGs, including *FOXO1*, *MAP1LC3C*, *CTSB*, *FOXO3*, *CALCOCO2*, *FKBP1A*, and 4 downregulated ARGs, including *MAPK8IP1*, *NRG1*, *VEGFA*, and *ITGA6* were consistent with the expression of high-throughput sequencing data.

**Conclusion:**

We identified 37 ARGs during osteogenic differentiation using bioinformatics analysis. FOXO1, MAP1LC3C, CTSB, FOXO3, CALCOCO2, FKBP1A, MAPK8IP1, NRG1, VEGFA, and ITGA6 may regulate osteogenic differentiation of hBMSCs by involving autophagy pathway. This study provides new insight into the osteogenic differentiation of hBMSCs and may be available in developing therapeutic strategies for maxillofacial bone defects.

## Introduction

Trauma, tumor resection, periodontitis and congenital disabilities often lead to severe maxillofacial bone defects (1-4). Finding effective means to repair bone defects to restore maxillofacial masticatory function, facial appearance, and mental health in patients is imperative. The process of bone tissue regeneration relies on the body’s recruitment of bone marrow mesenchymal stem cells (BMSCs) to the bone defect area, in concert with various growth factors, to promote their differentiation into osteoblasts (5, 6). The morphology of BMSCs is similar to that of fibroblasts, which is spindle-shaped, has the ability of adherent growth, can multiply and has the potential of multidirectional differentiation, and can be directionally differentiated into osteoblasts, chondrocytes, adipocytes, and neurons under specific conditions (7). BMSCs can undergo morpho-functional remodeling and differentiate into osteoblasts with the synergistic involvement of epigenetics, transcription factors, and autophagy-lysosome system pathways (8-10). In the biological processes above, autophagy plays a pivotal role in the osteogenic differentiation of BMSCs.

Macroautophagy (hereinafter referred to as “autophagy”) is a conserved endogenous intracellular degradation system widely found in eukaryotic cells (11, 12). Autophagosomes are vesicular structures with bilayer membranes, and their formation is mainly regulated by the ATGs family and other necessary proteins (13). Under conditions such as starvation, hypoxia, and endoplasmic reticulum stress, autophagosomes sequester cargos in the cytoplasm and deliver them to lysosomes for digestion to achieve energy recirculation (11, 13). A series of studies have shown that autophagy activation plays a vital role in the self-renewal potential and pluripotency of stem cells and is widely involved in the process of cell stability and differentiation (9, 14, 15). In the undifferentiated state of mesenchymal stem cells (MSCs), the autophagic pathway keeps the cells in a relatively stable phase of dynamic equilibrium by strictly limiting the operation of cytoplasmic proteins and organelles to maintain their multidirectional differentiation potential (13, 16). Meanwhile, autophagy is highly involved in the mineralization of MSCs and osteoblasts. Nollet *et al.* knocked down the autophagy-related genes (ARGs) *ATG7* and *BECLIN1* in osteoblast cell lines and showed a significant reduction in mineralized nodules in the knockdown group compared to the control (17). Similarly, rapamycin significantly enhanced the osteogenic differentiation of BMSCs by activating autophagy (18). However, the autophagy-related genes involved in the osteogenic differentiation of BMSCs are still unclear, and extensive validation is needed to find precise and efficient therapeutic targets for treating maxillofacial bone defects. 

The advent of high-throughput sequencing technologies has enabled bioinformatics databases like the Gene Expression Omnibus (GEO) and The Cancer Genome Atlas (TCGA) to emerge as practical tools for researchers to predict therapeutic targets for diseases. This study downloaded the high-throughput sequencing dataset GSE178679 from the GEO database to obtain gene expression profiles in human bone marrow mesenchymal stem cells (hBMSCs) osteo-induced and undifferentiated groups. R software was used to analyze the differentially expressed genes related to the osteogenic differentiation of hBMSCs. To explore the osteogenic differentiation process of hBMSCs from the perspective of autophagy, differentially expressed ARGs were obtained by intersecting ARGs from the Human Autophagy Database with differentially expressed genes. Gene Ontology (GO) enrichment analysis, Kyoto Encyclopedia of Genes and Genomes (KEGG) pathway analysis, and protein-protein interaction (PPI) network construction were used to speculate the possible annotations of differentially expressed ARGs and their interactions. Finally, RT-qPCR was used to verify the expression level of the differentially expressed ARGs in the hBMSCs osteo-induced samples and controls.

## Materials and Methods

***High-throughput sequencing data and autophagy-related genes database***

hBMSCs osteogenic differentiation high-throughput sequencing dataset GSE178679 was downloaded from Gene Expression Omnibus (http://www.ncbi.nlm.nih.gov/geo/, which was based on GPL20795 platform (Illumina HiSeq X Ten (Homo sapiens)), containing three osteogenic induction samples and three negative controls. Autophagy-related genes were screened from the Human Autophagy Database (http://www.autophagy.lu/index.html), and 222 genes were finally obtained. 

***Filtering differentially expressed ARGs***

The raw count value data was downloaded from the GSE178679 dataset, and the repeatability of the samples was analyzed using principal component analysis (PCA). Next, the “DEseq2” package of R software (version 4.0.5) was used to perform the differential expression analysis. Genes with a p-value <0.05 and absolute log2 fold-change value >0.5 were considered as differentially expressed genes. The obtained genes were then intersected with 222 ARGs to get the target genes. The volcano and PCA plots were generated using the R package “ggplot2”. Heatmap was generated using the R package “ComplexHeatmap”.

***Correlation analysis of the differentially expressed ARGs***

Correlation analysis and visualization of differentially expressed ARGs were performed using the “corrplot” package of R software, and the “ward.D” method was applied for hierarchical clustering.

***Gene ontology enrichment analysis and pathway enrichment analysis***

GO enrichment analysis and KEGG pathway analysis of differentially expressed ARGs were examined using the R package “clusterProfiler”. GO includes three domains: biological process (BP), molecular function (MF), and cellular component (CC). The enrichment analysis result with a *P*-value less than 0.05 was finally adopted.

***Protein-protein interaction network construction and hub gene mining***

The Search Tool for the Retrieval of Interacting Genes (STRING) (https://string-db.org/) database was used to construct a PPI network. Then, the PPI network was visualized by Cytoscape v3.8.0. The plugin Cytohubba in Cytoscape is based on integrating multiple network topology algorithms that can identify critical nodes and sub-networks in the PPI network. It was used to mine the hub genes in the constructed PPI network.

***Cell culture and osteo-induction***

The hBMSCs were purchased from Cyagen, China. The P3 hBMSCs were seeded into a 12-well plate at a density of 1.5×10^5 ^cells per well and cultured in low-glucose Dulbecco’s modified Eagle’s medium (Hyclone, USA) containing 10% fetal bovine serum (FBS), 100 U/ml penicillin and 100 μg/ml streptomycin at 37℃ in the presence of 5% CO_2_. The medium was renewed every three days. As described in the manufacturer’s manual, differentiation was induced by a 7-day culture in the osteogenic inductive medium (Cyagen Osteogenic Differentiation Kit). Mineralization was detected by staining with Alizarin red-S staining (Cyagen, China). Alkaline phosphatase staining was performed according to the manufacturer’s instructions (Alkaline Phosphatase Detection Kit, P0321S, Beyotime).

***Total RNA extraction and RT-qPCR***

The total RNA was extracted using RNAiso Plus (Takara, Japan) from the hBMSCs. Then, the Q5000 UV-Vis Spectrophotometer (Quawell, USA) instrument was used to detect the quality and concentration of total RNA. Reverse transcription was performed using the Evo M-MLV RT Kit(AG, China). The resulting cDNA, forward and reverse primers, and SYBR (Hieff® qPCR SYBR Green Master Mix, Yeasen, China) were used for RT-qPCR. The cycle threshold (C_t_) values for genes of interest were normalized against the C_t_ values of *GAPDH.* The relative mRNA expression was calculated using the 2ˆ(-ΔΔCT) method. Primer sequences are detailed in supplementary Table S1.

***Statistical analysis***

All results were independently repeated three times. Statistical analysis was performed using R software. Two-tailed Student’s t-test determined the statistical differences between the two groups, and *P*<0.05 was considered statistically significant. 

## Results

***Differentially expressed ARGs during osteogenic differentiation***

**Figure 1:**
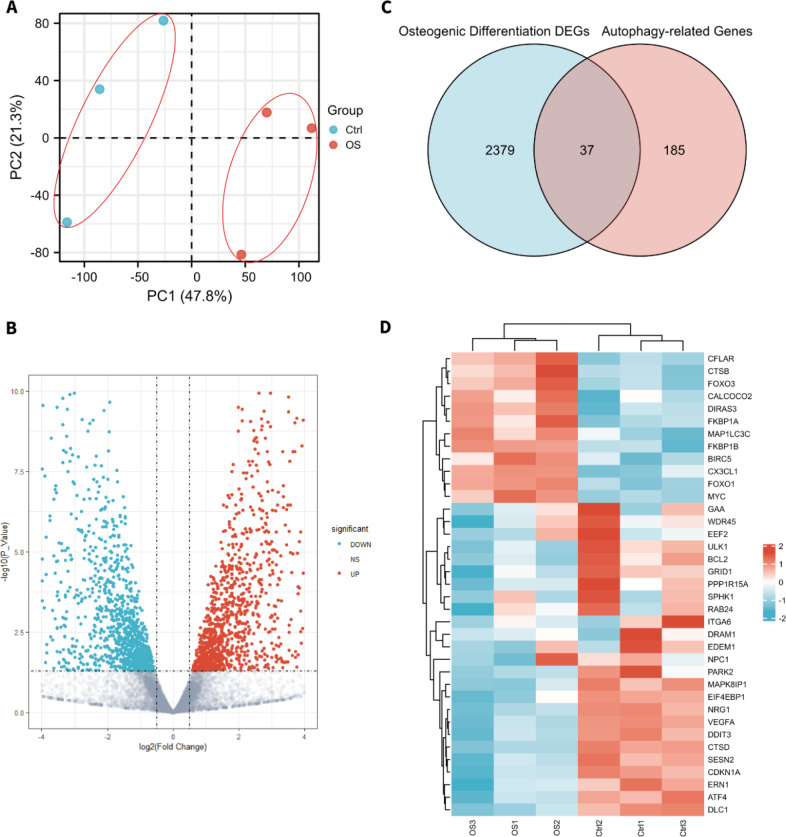
Differentially expressed ARGs in hBMSCs osteogenic differentiation dataset GSE178679. Red represents upregulated genes, and blue represents downregulated genes. A. the PCA plot of GSE178679. B. The volcano plot of the significantly expressed mRNA during osteogenic differentiation of hBMSCs. C. The Venn plot for screening differentially expressed ARGs. D. The heatmap of 12 upregulated and 25 downregulated differentially expressed ARGs

ARGs: Autophagy-related genes; hBMSCs: Human bone marrow mesenchymal stem cells; PCA: Principal component analysis

Repeatability and inter-group differences of the GSE178679 dataset were conducted using principal component analysis (PCA). The results indicate significant inter-group differences and repeatability in the dataset (Figure 1A). Then, the dataset GSE178679 was analyzed to explore the differentially expressed genes between the osteogenesis induction group and the control group. The results showed that 2416 differentially expressed genes were finally obtained, including 1231 downregulated and 1185 upregulated genes. The volcano plot for visualization of differentially expressed genes could be found in Figure 1B. 

**Figure 2:**
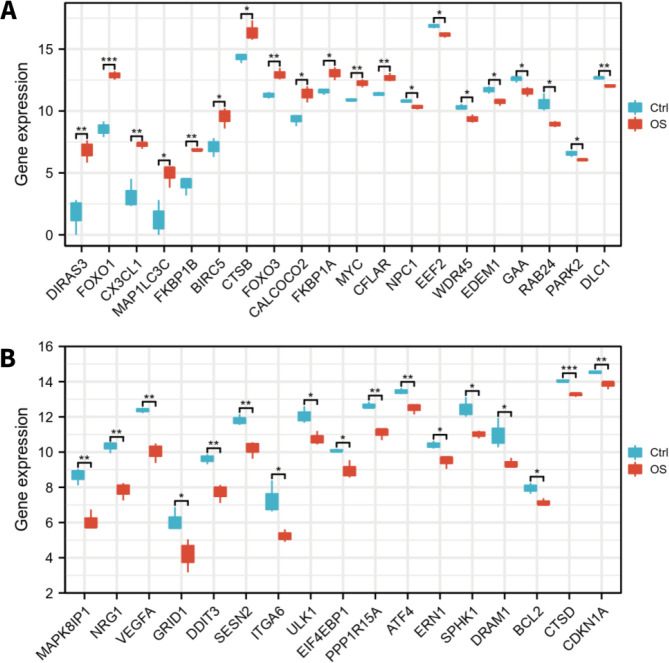
The boxplots of 37 differentially expressed ARGs during Osteogenic Differentiation of hBMSCs. A. The expression level of the top 20 significantly expressed ARGs in osteo-induced hBMSCs compared to controls. B. The last 17 significantly expressed ARGs in osteo-induced hBMSCs compared to controls

Venn analysis was conducted to examine the intersection among the differentially expressed genes and ARGs, and 37 target genes were finally identified, including 12 upregulated and 25 downregulated genes (Figure 1C and Table 1). The heatmap and boxplots were conducted to visualize the expression patterns of differentially expressed ARGs (Figure 1D, Figure 2A and B). The top ten upregulated ARGs included *DIRAS3*, *FOXO1*, *CX3CL1*, *MAP1LC3C*, *FKBP1B*, *BIRC5*, *CTSB*, *FOXO3*, *CALCOCO2*, *FKBP1A*, and the top ten downregulated ARGs included *MAPK8IP1*, *NRG1*, *VEGFA*, *GRID1*, *DDIT3*, *SESN2*, *ITGA6*, *ULK1*, *EIF4EBP1*, *PPP1R15A*.

***Delineation of GO and KEGG pathway analysis of differentially expressed ARGs***

**Figure 3:**
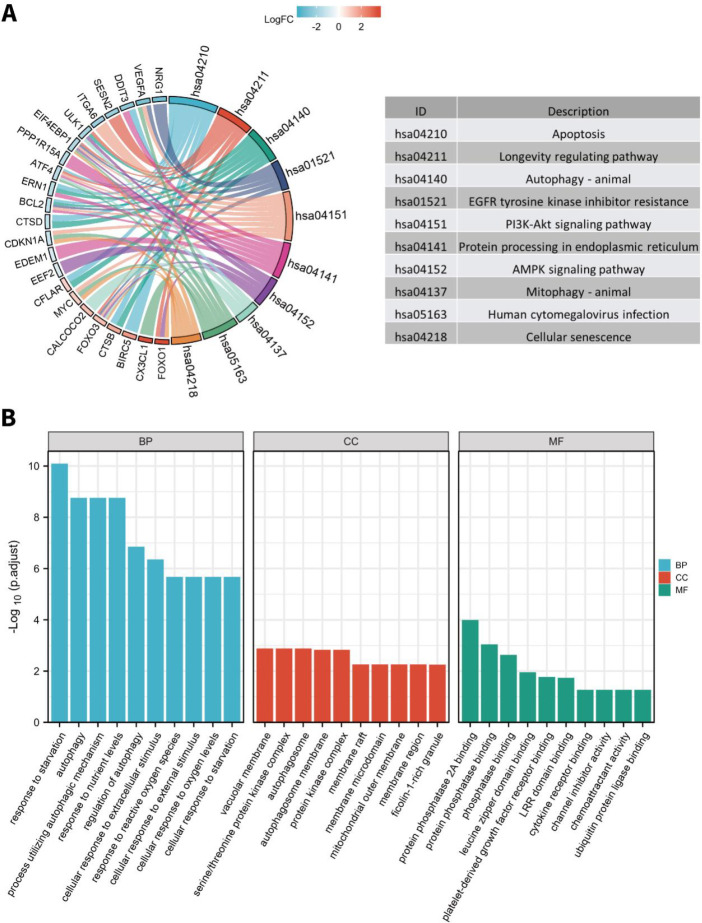
Pathway analysis and functional analysis of differentially expressed ARGs. A. KEGG pathway enrichment analysis of differentially expressed ARGs. B. GO Figure 4. The PPI network and hub gene identification. A. PPI network constructed by differentially expressed ARGs. B. The top 10 hub genes in the PPI network identified by the CytoHubba plugin. C. The interaction numbers of each differentially expressed ARG in the PPI network functional enrichment analysis of differentially expressed ARGs. BP: biological process. CC: cellular component. MF: molecular function

KEGG: Kyoto Encyclopedia of Genes and Genomes; ARGs: Autophagy-related genes; GO: Gene Ontology; PPI: Protein-protein interaction

The functional characteristics of the differentially expressed ARGs were obtained by performing GO enrichment analysis and KEGG pathway analysis. Figure 3 lists the top 10 enriched GO terms and KEGG pathways. Our data demonstrated that the biological processes related to the differentially expressed ARGs were markedly enriched in response to starvation, autophagy, and process utilizing autophagic mechanism. Regarding the cellular components, most of the differentially expressed ARGs were associated with the vacuolar membrane, serine/threonine protein kinase complex, the autophagosome, and the autophagosome membrane. Moreover, the molecular functions are mainly associated with protein phosphatase binding, leucine zipper domain binding, platelet-derived growth factor receptor binding, and cytokine receptor binding. The top five significantly enriched terms for KEGG pathway analysis were apoptosis, longevity regulating pathway, autophagy-animal, EGFR tyrosine kinase inhibitor resistance, and PI3K-Akt signaling pathway. The details are shown in supplementary Tables S2 and S3.

***Construction and analysis of the protein-protein interaction network***

**Figure 4:**
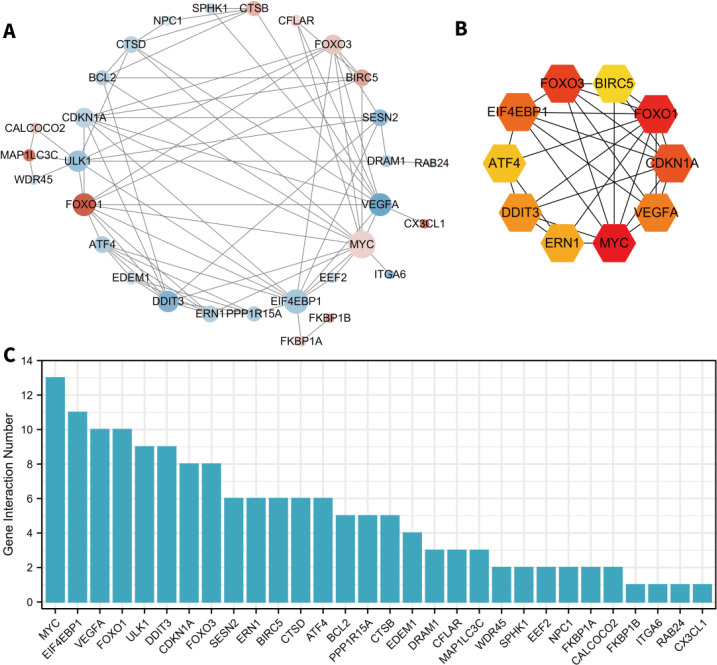
The PPI network and hub gene identification. A. PPI network constructed by differentially expressed ARGs. B. The top 10 hub genes in the PPI network identified by the CytoHubba plugin. C. The interaction numbers of each differentially expressed ARG in the PPI network

PPI: Protein-protein interaction; ARGs: Autophagy-related genes

**Figure 5:**
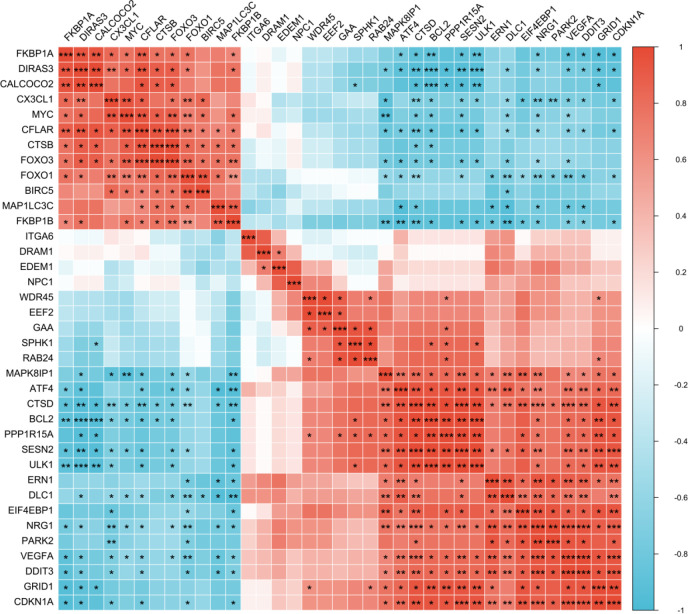
Spearman correlation analysis of 37 differentially expressed ARGs. ***: *P*< 0.001, **: *P*<0.01, *: *P*<0.05

We used the STRING database and Cytoscape software to test the potential protein interactions between the thirty-seven differentially expressed ARGs and construct a visualization network. The result indicated that 30 nodes (proteins) and 76 edges (interactions) were involved in the network (Figure 4A). The top ten hub gene mining was conducted using the “CytoHubba” plugin of Cytoscape, and the result is shown in Figure 4B. After the score ranking calculation, *MYC*, *FOXO1*, *FOXO3*, *CDKN1A*, *EIF4EBP1*, *VEGFA*, *DDIT3*, *ERN1*, *ATF4,* and *BIRC5* were the most connected genes in the PPI network. In addition, we analyzed the correlations between the 37 differentially expressed ARGs to explore their expression patterns, as shown in Figure 5.

***Validation of mRNA expression of differentially expressed ARGs in hBMSCs***

**Figure 6:**
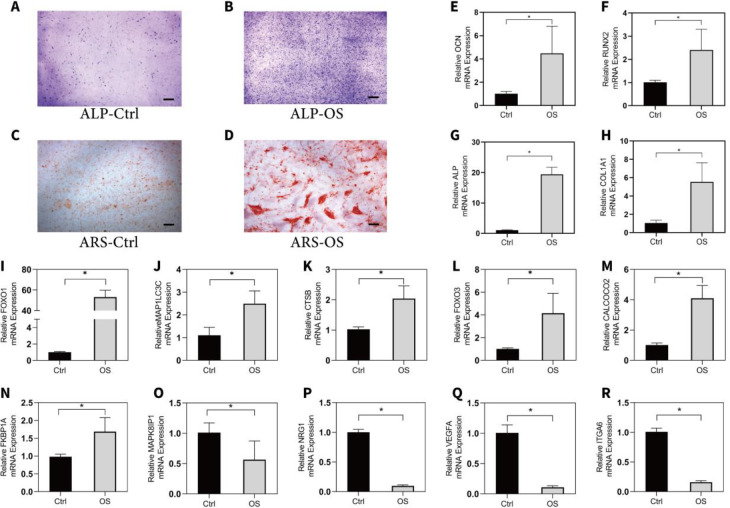
The mRNA expression level of the top 20 differentially expressed ARGs and osteogenic makers were validated by RT-qPCR in osteo-induced hBMSCs and controls. A-B. Alkaline phosphatase staining between osteo-induced hBMSCs and controls. C-D, Alizarin Red S staining between osteo-induced hBMSCs and controls. E-H. The osteogenic makers were markedly upregulated after osteogenic induction. I-N. Among the upregulated differentially expressed ARGs, FOXO1, MAP1LC3C, CTSB, FOXO3, CALCOCO2, and FKBP1A were consistent with the expression of sequencing data. O-R. Among the downregulated differentially expressed ARGs, MAPK8IP1, NRG1, VEGFA, and ITGA6 were consistent with the expression of sequencing data. P-values were calculated using the two-tailed Student’s t-test. *P<0.05. Scale bar=200 um

ARGs: Autophagy-related genes; hBMSCs: Human bone marrow mesenchymal stem cells

**Figure 7:**
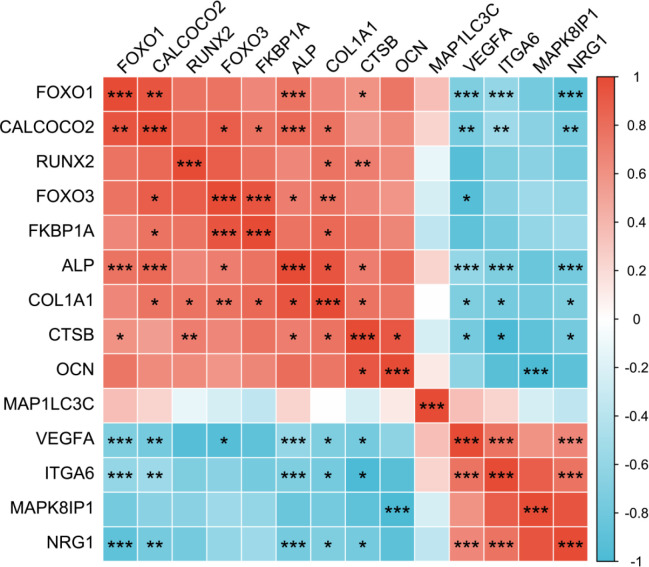
The Spearman correlation analysis between the expression level of identified ARGs and osteogenic markers. ***P< 0.001, **P< 0.01, *P< 0.05

We selected the top twenty differentially expressed upregulated and downregulated ARGs and explored their expression levels during the osteogenic differentiation process of hBMSCs. The mRNA expression level of osteogenic markers, including *ALP*, OCN, *RUNX2*, and *COL1A1* was detected between the osteo-induced and undifferentiated groups. The results revealed that among the differentially upregulated ARGs, *FOXO1*, *MAP1LC3C*, *CTSB*, *FOXO3*, *CALCOCO2*, and *FKBP1A* were consistent with the expression of sequencing data. Among the differentially downregulated ARGs, *MAPK8IP1*, *NRG1*, *VEGFA*, and *ITGA6* were also downregulated in the osteogenic differentiation group of the sequencing data (Figure 6). The correlations between these identified ARGs and osteogenic markers are shown in Figure 7. 

The osteogenic makers were markedly upregulated after osteogenic induction. I-N. Among the upregulated differentially expressed ARGs, *FOXO1*, *MAP1LC3C*, *CTSB*, *FOXO3*, *CALCOCO2*, and *FKBP1A* were consistent with the expression of sequencing data. O-R. Among the downregulated differentially expressed ARGs, *MAPK8IP1*, *NRG1*, *VEGFA*, and *ITGA6* were consistent with the expression of sequencing data. *P*-values were calculated using the two-tailed Student’s t-test. *P<0.05. Scale bar=200 um.

**Table 1:** The thirty-seven differentially expressed Autophagy-related genes (ARGs) in osteo-induced Human bone marrow mesenchymal stem cells hBMSCs compared to controls

Gene Symbol	logFC	Regulation	P.value	Location
*DIRAS3*	4.725723	Up	9.96E-09	1p31.3
*FOXO1*	3.726202	Up	4.08E-17	13q14.11
*CX3CL1*	3.409896	Up	1.48E-06	16q21
*MAP1LC3C*	3.225293	Up	0.0087	1q43
*FKBP1B*	2.332649	Up	9.83E-05	2p23.3
*BIRC5*	1.931086	Up	0.00087	17q25.3
*CTSB*	1.613988	Up	4.31E-05	8p23.1
*FOXO3*	1.188687	Up	9.71E-05	6q21
*CALCOCO2*	1.174285	Up	0.008974	17q21.32
*FKBP1A*	0.981605	Up	0.002556	20p13
*MYC*	0.888196	Up	0.011425	8q24.21
*CFLAR*	0.832867	Up	0.002336	2q33.1
*NPC1*	-0.63726	Down	0.026999	18q11.2
*EEF2*	-0.78436	Down	0.034471	19p13.3
*WDR45*	-0.8013	Down	0.022832	Xp11.23
*EDEM1*	-0.8066	Down	0.006045	3p26.1
*GAA*	-0.90057	Down	0.02849	17q25.3
*RAB24*	-0.9597	Down	0.0351	5q35.3
*PARK2*	-1.00779	Down	0.025438	6q26
*DLC1*	-1.09825	Down	0.000223	8p22
*CDKN1A*	-1.169	Down	0.000347	6p21.2
*CTSD*	-1.27681	Down	8.76E-05	11p15.5
*BCL2*	-1.29799	Down	0.003806	18q21.33
*DRAM1*	-1.3662	Down	0.014844	12q23.2
*SPHK1*	-1.37272	Down	0.030133	17q25.1
*ERN1*	-1.38131	Down	3.13E-05	17q23.3
*ATF4*	-1.43917	Down	3.45E-05	22q13.1
*PPP1R15A*	-1.47155	Down	0.000701	19q13.33
*EIF4EBP1*	-1.61701	Down	7.53E-07	8p11.23
*ULK1*	-1.81044	Down	9.54E-05	12q24.33
*ITGA6*	-2.08392	Down	0.008852	2q31.1
*SESN2*	-2.09377	Down	4.63E-07	1p35.3
*DDIT3*	-2.33435	Down	1.07E-08	12q13.3
*GRID1*	-2.39011	Down	0.001144	10q23.1-q23.2
*VEGFA*	-2.77087	Down	2.62E-12	6p21.1
*NRG1*	-2.95384	Down	1.42E-15	8p12
*MAPK8IP1*	-3.16563	Down	3.37E-12	11p11.2

ARGs: Autophagy-related genes; hBMSCs: Human bone marrow mesenchymal stem cells

ARGs: Autophagy-related genes

ARGs: Autophagy-related genes

## Discussion

The oral and maxillofacial region is closely related to critical physiological functions such as swallowing, speech, mastication, and appearance. However, tumors, trauma, and other factors can lead to severe defects in the maxillofacial bone that are difficult to heal spontaneously. However, the mechanism of regulating osteogenic differentiation is not completely clear, and more comprehensive exploration is still needed to deepen the understanding of osteogenic differentiation and promote the accurate treatment of bone defect diseases. BMSCs are multipotent stem cells with self-renewal capacity and multilineage differentiation potential significantly enriched in the maxillofacial skeleton and derived from mesoderm. Their stemness maintenance and osteogenic differentiation are controlled by various factors, including transcription factors, secreted cytokines, and cell interactions (14, 19, 20). A growing number of studies have found that autophagy plays an essential role in this process. MSCs maintain a relatively static dynamic equilibrium in the undifferentiated state, where cells regulate the intracellular recycling process of organelles and proteins through autophagy activation to maintain their pluripotency and plasticity (14, 21). In the process of cell differentiation, autophagy activation can provide the energy and nutrients needed for transforming cell morphology and function and regulate the operation of stem cell remodeling into osteoblasts.

In recent years, with the development of high-throughput sequencing technology in the field of molecular biology, it has been found that the expression of many RNA molecules has changed significantly during the osteogenic differentiation of BMSCs. By transcriptome sequencing, Xu *et al*. performed a transcriptomic analysis of osteogenic induction and control groups of BMSCs. Their study identified several significantly differentially expressed genes during osteogenic differentiation, including lncRNA LOC100126784, lncRNA POM121L9P, miR-503-5p, and *SORBS1* (22). Others reported that linc-ROR was significantly upregulated while miR-138 and miR-145 were downregulated during the osteogenic differentiation of BMSCs. linc-ROR could act as a molecular sponge to antagonize the function of these two miRNAs, thereby derepressing their common target ZEB2 and ultimately activating the Wnt/β-catenin signaling pathway to promote osteogenic differentiation (23). In addition, it has been found that under H_2_O_2_-induced oxidative stress, the autophagy-lysosome system could degrade the osteogenic key factor TP53INP2 and thus inhibit the osteogenic differentiation of BMSCs (24).

The exploration of autophagy brings a new weathervane for treating bone defect diseases. However, the current research on autophagy involved in the osteogenic differentiation of BMSCs only focuses on a single signal axis mode. The emergence of high-throughput sequencing technology has enabled people to study the regulatory process of genes from a new perspective. In this study, we first applied bioinformatics analysis to identify thirty-seven differentially expressed ARGs during osteogenic differentiation of BMSCs by searching GEO and the Human Autophagy Database. To explore the possible functions and pathways involved in these differentially expressed ARGs, we performed GO and KEGG enrichment analysis, which showed that most of these genes were enriched to the terms of apoptosis, longevity regulating pathway, and autophagy. Recently, scholars have published many studies to verify the involvement of autophagy in the osteogenic differentiation of MSCs. It has been shown that in gingival-derived MSCs, the autophagy activator resveratrol and osteogenic-inducing factor act together on the pro-autophagic pathway AMPK-BECLIN1 to induce osteogenic differentiation. However, after promoting proteasome-mediated degradation of BECLIN1 using the autophagy inhibitor Spautin-1, HGMSCs exhibited a poor osteogenic state compared to the controls despite the presence of resveratrol and osteogenic-inducing factors (25). Similarly, in IL-1β induced inflammatory conditions, GABARAP can promote osteogenic differentiation of BMSCs by increasing autophagic activity and reducing ROS production (26). However, broader validation is needed to improve the understanding of the mechanisms of the autophagy process in the osteogenic differentiation of BMSCs.

In this study, we selected the top ten up and downregulated differentially expressed ARG to further validate *in vitro* by RT-qPCR. The results showed *FOXO1*, *MAP1LC3C*, *CTSB*, *FOXO3*, *CALCOCO2*, *FKBP1A*, *MAPK8IP1*, *NRG1*, *VEGFA,* and *ITGA6* were consistent with the sequencing data, and the difference was statistically significant. Furthermore, correlation analysis between the expression levels of identified ARGs and osteogenic markers suggests a possibly complex regulatory role for these ARGs in osteogenic differentiation. Some published articles explore the potential mechanisms of these genes. *FOXO1* and *FOXO3* are members of the forkhead family, key transcription factors that can regulate the osteogenic differentiation of BMSCs in the early stage (27-29). MAP1LC3C is rarely reported in the osteogenic differentiation process, but it can participate in the odontogenic differentiation of human dental pulp stem cells by regulating autophagy flow (30). Yang et al. reported that the SNP rs2736308 on chromosome 8 could regulate the expression of CTSB and then interact with EGFR to downregulate jaw bone mineral density in medication-related osteonecrosis of the jaw (31). Another study revealed that compared to adult individuals, periodontal ligament stem cells from young subjects have higher expression levels of FKBP1A and osteogenic markers (32). It has been found that overexpression of hsa_circ_0006215 increased the expression level of VEGFA, thus promoting the formation of bone-specific H-type vessels to enhance the osteogenic differentiation of BMSCs (33). However, the specific mechanism of the autophagy pathway involved in these genes to regulate the osteogenic differentiation of BMSCs remains unclear and needs to be further explored.

Current studies have also reported the involvement of autophagy in the development of different orthopedic diseases. A recent study demonstrated that quercetin and vitamin E could relieve osteoporosis in ovariectomized rats by regulating the autophagy pathway (34). Similarly, systemic use of rapamycin can induce osteocyte autophagy, thereby alleviating osteoporosis in older rats (35). It has been found that the autophagy level of chondrocytes in osteoarthritis rats can be increased by inhibiting PI3K/AKT/mTOR signal pathway, thus reducing the inflammation of the body (36). Therefore, autophagy plays a vital role in osteogenic differentiation and orthopedic diseases.

Our study has several limitations. First, the sample size we included in this study was small, and more samples are needed to exclude possible false-positive results. Second, the *in vitro* experiment of this study stays at the verification stage of gene expression level. We will next conduct a more in-depth study on the specific mechanism of autophagy in the process of osteogenic differentiation in the future in order to provide accurate therapeutic targets for the reconstruction of bone defects.

## Conclusion

In summary, our study identified thirty-seven ARGs during osteogenic differentiation using bioinformatics analysis. *FOXO1*, *MAP1LC3C*, *CTSB*, *FOXO3*, *CALCOCO2*, *FKBP1A*, *MAPK8IP1*, *NRG1*, *VEGFA*, and *ITGA6* may regulate osteogenic differentiation of hBMSCs by involving autophagy pathway. This study provides new insight into the osteogenic differentiation of hBMSCs and may be available in developing therapeutic strategies for maxillofacial bone defects.

## Authors’ Contributions

DW, YL and XY Designed the experiments; YL, XY, YFX, CWL, JHX Performed the experiments and collected data; YL, XY and YJL Discussed the results and strategy; DW Supervised, directed and managed the study; YL, XY and DW Final approved of the version to be published; DW was responsible for funding acquisition and project administration.

## Conflicts of Interest

The authors declare no conflicts of interest associated with the present manuscript.
